# Drop-out and mood improvement: a randomised controlled trial with light exposure and physical exercise [ISRCTN36478292]

**DOI:** 10.1186/1471-244X-4-22

**Published:** 2004-08-11

**Authors:** Sami Leppämäki, Jari Haukka, Jouko Lönnqvist, Timo Partonen

**Affiliations:** 1 Department of Mental Health and Alcohol Research, Unit for Epidemiology and Genetics of Mental Health, National Public Health Institute, Helsinki, Finland; 2 Department of Psychiatry, Jorvi Hospital, Helsinki University Central Hospital, Espoo, Finland

**Keywords:** phototherapy, bright light, seasonal, depressive symptoms, exercise

## Abstract

**Background:**

Combining bright light exposure and physical exercise may be an effective way of relieving depressive symptoms. However, relatively little is known about individual factors predicting either a good response or treatment failure. We explored background variables possibly explaining the individual variation in treatment response or failure in a randomised trial.

**Methods:**

Participants were volunteers of working-age, free from prior mental disorders and recruited via occupational health centres. The intervention was a randomised 8-week trial with three groups: aerobics in bright light, aerobics in normal room lighting, and relaxation/stretching in bright light. Good response was defined as a 50% decrease in the symptom score on either the Hamilton Depression Rating Scale (HDRS) or 8-item scale of atypical symptoms. Background variables for the analysis included sex, age, body-mass index, general health habits, seasonal pattern, and sleep disturbances.

**Results:**

Complete data were received from 98 subjects (11 men, 87 women). Of them, 42 (5 men, 37 women) were classified as responders on the HDRS. Overall, light had a significant effect on the number of responders, as assessed with the HDRS (X^2 ^= .02). The number needed to treat (NNT) for light was 3.8.

**Conclusions:**

We investigated the effect of bright light and exercise on depressive symptoms. Problems with sleep, especially initial insomnia, may predict a good response to treatment using combined light and exercise. Bright light exposure and physical exercise, even in combination, seem to be well tolerated and effective on depressive symptoms.

## Background

At northern latitudes reduced vitality, increased appetite and sleep complaints are common symptoms during wintertime, even among people considered healthy [1]. Atypical depressive symptoms, which are often seen in seasonal depression, appear to correlate with decreased illumination [2]. Exposure to natural light also appears to have a substantial effect on well-being in twins with bipolar disorder [3]. Disorganised circadian clockwork, related to the shortening photoperiod and changes in this most important external time-giver, is thought to play a role in the pathophysiology of seasonal mood changes. Bright (>2500 lx) light therapy has proven effective for season-related major depressive episodes, and also for milder, subsyndromal symptoms [4]. An interesting alternative to bright light is dawn simulation (short, timed pulses of light during the natural awakening), which may be helpful in seasonal affective disorder [5] and sleeping problems, even in the general population [6].

Exercise would seem to be the ideal treatment for depression: available, affordable, with minimal side effects. Unfortunately, many exercise treatment studies suffer from methodological weaknesses and lack of adequate follow-up to determine long-term efficacy. In their systematic review, Lawlor and Hopker even concluded that the effectiveness of exercise in reducing depressive symptoms cannot be determined because of a lack of good quality research, though exercise was found to be more effective than no treatment, and as effective as cognitive therapy [7]. Fortunately, absence of evidence does not necessarily indicate evidence of absence. A recent 16-week study with rigorous methodology indicates that exercise is as effective as standard medication (sertraline) for treatment of depression [8]. A 6-month follow-up of the study subjects showed that remitted subjects in the exercise group had significantly lower relapse rates than remitted subjects in the medication group, and that continued exercise was associated with lower rates of depression [9]. Other studies on the efficacy of exercise treatment for depression are also conducted, to address e.g. the question of a dose-response relationship [10]. Several mechanisms may explain the mood-lifting effects of exercise: psychological (increased sense of self worth, positive feedback), social (an increase in social contacts), and physiological (changes in central endorphin and monoamine concentrations). Exercise may induce phase-shifts in the human circadian rhythms [11], so it is possible that exercise also may exert part of its action on mood by influencing the circadian clock.

Adding light exposure to exercise for the treatment of depression and depressive symptoms seems a promising intervention. Combinations of bright light exposure and physical exercise have achieved beneficial effects on mood in trials on healthy adult populations [12,13]. Natural light exposure (one hour walk outdoors) may also be effective in treating seasonal affective disorder [14].

It remains to be determined, however, whether it is possible to define a subgroup of people who are especially likely to benefit from this kind of intervention, and what, if any, are the individual factors predicting a good response to light or exercise, or their combination? And on the contrary, another important subgroup to identify, in determining the efficacy of any intervention, is the subjects who drop out of the study prematurely. Several predictors of response to light treatment in winter depressives have been identified: the ratio of atypical to classical symptoms of depression [15] and hypersomnia, increased eating and younger age [16]. Meesters et al. found that a large diurnal variation had a negative predictive value on response to light treatment [17]. Temperament dimensions have also been investigated. Although higher harm avoidance scores have been linked to non-response to light therapy in one study [18], another study found no predictive value of avoidance scores [19]. With regard to exercise treatment of depression, baseline levels of self-reported anxiety and life satisfaction were found to be best predictors of both dropout and treatment success, when exercise alone was compared to antidepressive medication or exercise with medication [20].

In the present study we tried to address this question: who will benefit from light, exercise or their combination? A variety of background variables were investigated: age, seasonality, depressive and atypical symptoms, treatment adherence, fitness, body mass index, self-perceived quality of sleep, and alcohol consumption. We did not measure diurnal variation or personality dimensions.

## Methods

Adult volunteers of working age were invited to participate in a study of light and exercise via occupational health centres. The enrolled subjects were randomly allocated to three intervention groups: aerobics training in bright light (>2500 lx, measured at eye-level), similar training in the normal lighting of the gym (400–600 lx), and relaxation and stretching sessions in bright light. The training or relaxation sessions (45 minutes each, starting at 7:30 a.m. or 8:30 a.m. Monday through Friday, and at 10:00 a.m. or 11:00 a.m. on Saturdays) were scheduled two times a week over eight weeks. Study was conducted between November 25, 1997, and January 25, 1998. The length of daylight on these dates was 6 h 48 min and 7 h 23 min, respectively.

### Intervention

Both the aerobics training and relaxation groups were led by 3 physiotherapists, each of whom supervised one third of each group's training or relaxation sessions. The training and relaxation sessions were structured to maintain treatment consistency. In the aerobics groups, the intensity of the training was checked with a heart rate monitor, the target rate being 120 to 150 beats per minute. Stretching/relaxation training was designed to avoid raising the pulse. All training sessions were in the same gym, the ceiling of which was equipped with 30 extra light fixtures with cool-white (6000 K) fluorescent lamps (F58W/186, Sylvania, Germany), which were turned on for the bright light groups.

### Assessment

Mood during the study period was recorded using the Structured Interview Guide for the Hamilton Depression Rating Scale – Seasonal Affective Disorders Version Self-Rating Format (SIGH-SAD-SR) [21], which includes the 21-item Hamilton Depression Rating Scale (HDRS) plus an eight-item addendum for atypical symptoms (ATYP). The SIGH-SAD-SR was filled in at baseline, after week 4 and at the end of the 8-week study period.

At the start of the study and at weeks 4 and 8, all subjects were weighed to assess body-mass index (BMI). Before and after the study period all subjects in the Aerobics training groups participated in a 2-km walking test, which predicts maximal oxygen uptake using a model with age, sex, walking time, BMI, and heart rate at the end of the test as variables [22]. At baseline, sleep quality was assessed with the Basic Nordic Sleep Questionnaire (BNSQ) [23], and the subjects also completed an abbreviated, 26-item FINRISK questionnaire [24] concerning smoking, alcohol consumption, dietary fat intake, and habitual exercise. The Seasonal Pattern Assessment Questionnaire (SPAQ) [25] measures seasonal changes in mood and behaviour. The SPAQ includes a 6-item scale yielding the Global Seasonality Score (GSS). Based on the GSS the subjects were thereby divided into seasonals and non-seasonals, according to the criteria for subsyndromal seasonal affective disorder presented by Bartko and Kasper [26]. Baseline demographic data on all participants included sex, age, and educational level.

### Ethics

All subjects returned a written informed consent prior to participation. The ethics committee of the National Public Health Institution approved the study protocol.

### Statistics

All analyses were done on SPSS for Windows (Release 11.5.1)-statistical package (SPSS Inc., Chicago, Illinois).

### Dropouts

Participants were classified as dropouts if they, for any reason, did not finish the eight-week study protocol. Analysis of variance (ANOVA)-models were used to compare the baseline characteristics of dropouts with those who completed the study. In the models, the baseline characteristic was the dependent variable, and the outcome (dropouts vs. completers) and treatment group (Light & Exercise, Exercise, Light) were factors. The Outcome × Treatment group interaction was also tested in each model. Background characteristics examined were age, GSS, BMI, fitness, and alcohol consumption, all as continuous variables. Baseline HDRS and ATYP were included, as well as percentage of sessions attended. From the BNSQ, following variables were chosen: initial, middle, and late insomnia, and quality of sleep.

Treatment benefit was defined separately as response and, with stricter criteria, as remission.

### Treatment response

The main outcome measures were changes in the HDRS, ATYP and the SIGH-SAD-SR over the 8-week study period. A 50 % decrease on the HDRS, ATYP or the SIGH-SAD-SR total score was used to divide subjects into responders and non-responders. To assess the effect of baseline characteristics, logistic regression models were formulated, with the defined clinical response as the dependent variable. Light therapy, physical exercise, and sex were constant in the models. Other independent baseline variables in the analysis were age (over/under 40 years), seasonality (from the SPAQ), initial, middle and late insomnia, quality of sleep, feeling tired after waking (from the BNSQ), serum total cholesterol levels, current smoking, physical training, and consumption of alcoholic beverages (from the FINRISK questionnaire). All categorical variables were dichotomised for the analysis. The best-predicting co-variates were found by backward step-wise selection. Analysis of variance (ANOVA) was applied to compare means between groups, and associations were analysed by calculating partial correlation coefficients, after controlling for age and sex. Pearson chi-square (two-sided) was used when applicable.

### Remission

Stricter criteria were applied for the definition of remission. To increase clinical meaningfulness and to avoid 'flooring effect', subjects with low symptom scores were excluded from these analyses.

#### HDRS

Only subjects with a baseline HDRS of eight or higher were included. Remission was defined as at least a 50% reduction on the HDRS during the trial, *and *a score of less than eight at the end of the study period.

#### SIGH-SAD

Subjects with a baseline SIGH-SAD-total score of fourteen or more were included. Remission was defined as at least a 50% reduction on the score during the eight-week study, *and *a final SIGH-SAD-score of eight or less.

All analyses were done 'intention-to-treat', i.e. dropouts were classified as treatment failures. With remitted subjects, we desisted from using logistic regression because of lower number of subjects, which would limit the number of variables. Instead, the background variables were examined one-by-one with ANOVA-models (see *Dropouts *for description).

## Results

**Figure 1:**
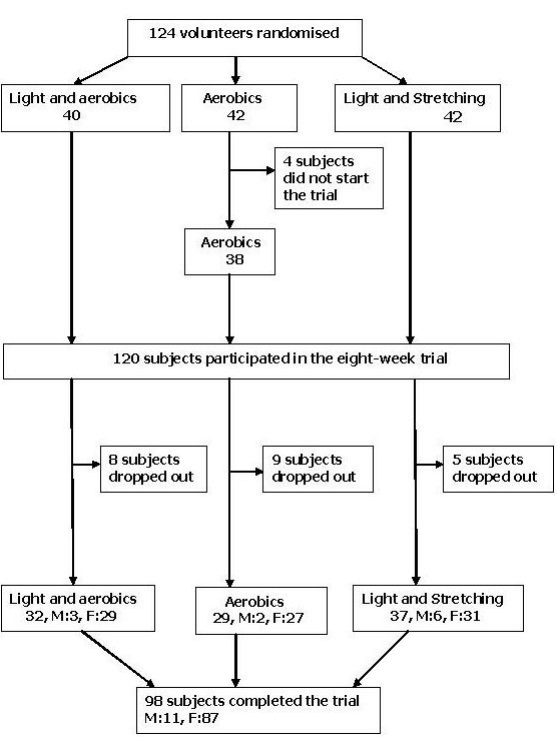
Study protocol

Complete data were received from 98 subjects (11 men, 87 women, see Figure 1) with a mean (s.d.) age of 43.4 (9.5), ranging from 26 to 63 years. Sixty-nine subjects were assigned to the light therapy groups, and 61 subjects to the aerobic exercise treatment groups. There were 37 of these subjects in the combined group, and their mean (s.d.) score on the HDRS was 10.5 (6.3), on the ATYP 5.9 (4.2), and on the SIGH-SAD-SR 16.4 (9.4). On average (s.d.), the GSS was 10.5 (4.9), and the BMI 24.2 (3.7). At baseline, the GSS was negatively associated with habitual training (r = -.26, p = .01), and correlated with initial insomnia (r = .20, p = .05), low quality of sleep (r = .30, p = .003), and feeling tired after waking (r = .35, p = .001). Subjects reporting initial insomnia on the BNSQ (n = 28) had, as expected, a higher score on the HDRS at baseline than other subjects (F = 11.2, p = 0.001), and they also had a higher ATYP score (F = 6.70, p = 0.01) and a trend towards higher GSS (F = 3.92, p = 0.05). Twenty-three of these subjects received light therapy, and 13 (57%) of them were classified as responders based on the changes in HDRS scores (X^2 ^= .04).

There was a negative correlation between response on the HDRS and alcohol consumption (more than 7 drinks a week) (r = -.23, p = .03), and high levels of serum total cholesterol (r = -.21, p = .04).

### Treatment response

Based on the HDRS, 42 subjects (5 men, 37 women, X^2 ^= .9) were classified as responders. Their mean age (s.d.) was 41.3 (9.5) years, ranging from 26 to 58. Thirty-five (83%) had had light therapy, 24 (57%) had been in the aerobic exercise groups, and 17 subjects (40%) in the combined group. Overall, light had a significant effect on the number of responders, as assessed with the HDRS (X^2 ^= .02). The number needed to treat (NNT) for light was 3.8.

On the basis of the ATYP scores, 51 subjects (8 men, 43 women) were classified as responders. Their mean age (s.d.) was 41.9 (9.8) years, ranging from 26 to 63. Thirty-seven (73%) had received light therapy, 30 (59%) had been in the exercise groups, and 16 (31%) in the combined group.

Response on the SIGH-SAD-SR was negatively associated with baseline self-reported alcohol consumption (r = -.26, p = .01). There were 45 responders (5 men, 40 women) on the SIGH-SAD-SR, with a mean age (s.d.) of 41.5 (9.5) years, ranging from 26 to 58. Thirty-seven (82%) had received light therapy, 26 (58%) had done aerobic exercise, and 18 (40%) had been in the combined group. The effect of light therapy was significant (X^2 ^= .03). Based on these figures, the NNT for light was 3.8.

The logistic regression models for the HDRS, ATYP, and SIGH-SAD-SR total scores are presented in Table 1.

**Table 1:** Key results from logistic regression models.

Variable	coefficient	s.e.	p value
***HDRS***			
Age group*	-.8	.50	.09
Total cholesterol**	-1.6	.66	.01
Alcohol consumption†	-1.6	.62	.008
***ATYP score***			
Age group*	-1.0	.52	.05
Initial insomnia‡	1.8	.72	.01
Quality of sleep ±	-2.1	.69	.002
Alcohol consumption†	-1.2	.60	.04
***SIGH-SAD-SR total score***			
Age group*	-1.1	.53	.04
Initial insomnia‡	1.5	.70	.03
Quality of sleep ±	-1.6	.66	.02
Alcohol consumption†	-1.8	.62	.005

*Age group: over 40 years vs. under 40 years **Total cholesterol: previous high total serum cholesterol, yes vs. no †Alcohol consumption: over 7 drinks per week vs. 7 drinks or less per week ‡Initial insomnia: trouble falling asleep once a week to daily vs. less than once week ± Quality of sleep: bad or rather bad vs. normal to good

### Dropouts

There were 26 (21%) subjects classified as dropouts (4 male, 22 female, X^2 ^= .6), 8 (20%) in the light & exercise group, 13 (31%) in the exercise group, and 5 (12%) in the light group (X^2 ^= .1). Thirteen subjects (50%) had received light therapy (X^2 ^= .05). Table 2 presents the background variables investigated from all subjects and by treatment group. Dropout-status was significantly influenced by the GSS (F = 5.40, p = .02) and treatment sessions attended (F = 143.0, p = .000). There was a trend towards baseline HDRS having an effect on dropout-status (F = 4.00, p = .05), but baseline HDRS was also influenced by treatment group (F = 4.38, p = .02), despite random assignment to groups. The pre-intervention fitness test result (in the exercise groups) was predictive of dropout status (F = 11.1, p = .001), and there was also a significant Treatment group × Dropout interaction (F = 7.82, p = .007). Initial insomnia, derived from the BNSQ, was also a significant factor (F = 6.00, p = .02, Treatment group × Dropout interaction F = 5.43, p = .006).

**Table 2:** Comparison of baseline variables (S.D) of drop-outs vs. completers

							***Analysis of variance***			
**Variable**					*Treatment group*		*Dropout group*		*Interaction*	

	**All**	**Exercise&Light**	**Exercise**	**Light**	**F**	**p**	**F**	**p**	**F**	**p**

**Age**					.50	.61	2.33	.13	2.20	.12
Drop-outs	39.5 (8.2)	41.3 (7.2)	35.4 (7.2)	43.4 (9.6)						
Completers	43.4 (9.6)	41.8 (9.2)	45.5 (10.2)	43.2 (9.3)						
**GSS**					1.35	.26	5.40	**.02**	.98	.38
Drop-outs	13.1 (4.5)	13.0 (5.0)	11.1 (3.3)	15.8 (4.5)						
Completers	10.5 (4.9)	10.1 (5.2)	10.5 (4.6)	10.9 (5.1)						
**HDRS**					4.38	**.02**	4.00	.05	1.55	.22
Drop-outs	13.6 (8.4)	16.3 (10.9)	8.29 (3.9)	16.6 (5.6)						
Completers	10.5 (6.4)	10.8 (7.0)	9.00 (5.2)	11.4 (6.6)						
ATYP*					1.18	.31	2.42	.12	.23	.79
Drop-outs	7.45 (5.3)	7.50 (5.4)	6.14 (5.0)	9.20 (6.0)						
Completers	5.92 (4.3)	6.22 (4.9)	5.07 (3.7)	6.32 (4.2)						
BMI**					.30	.74	1.88	.17	1.33	.27
Drop-outs	25.5 (4.9)	27.0 (6.2)	24.9 (4.5)	24.4 (3.8)						
Completers	24.2 (3.7)	23.6 (3.1)	24.1 (3.6)	24.7 (4.3)						
**Sessions attended (%)**					2.70	.07	143	**.000**	2.29	.11
Drop-outs	32.5 (24.4)	25.8 (20.6)	30.6 (24.9)	48.0(27.2)						
Completers	82.1 (14.4)	82.7 (14.4)	80.5 (15.2)	82.9(14.1)						
**Fitness**					2.61	.11	11.1	**.001**	7.82	**.007**
Drop-outs	93.3 (14.3)	83.7 (11.3)	99.1 (13.0)	----						
Completers	103.2 (12.1)	105.1 (13.6)	101.0 (10.2)	---						
**BNSQ**										
*Initial insomnia*					3.00	.06	6.00	**.02**	5.43	**.006**
Drop-outs	2.61 (1.2)	3.50 (.84)	2.14 (1.1)	2.20 (1.3)						
Completers	2.06 (.93)	1.84 (.81)	1.83 (.93)	2.43 (.93)						
*Middle insomnia*					1.91	.15	.23	.64	1.34	.27
Drop-outs	3.21 (1.4)	3.57 (1.4)	2.43 (1.1)	3.80 (1.6)						
Completers	3.11 (1.3)	3.10 (1.3)	3.03 (1.3)	3.19 (1.4)						
*Late insomnia*					2.97	.06	.55	.46	1.15	.32
Drop-outs	1.68 (.95)	1.86 (1.1)	1.14 (.38)	2.20 (1.1)						
Completers	1.92 (.92)	1.78 (.83)	1.79 (.90)	2.14 (.98)						
*Quality of sleep*					1.615	.20	.10	.753	.70	.50
Drop-outs	2.47 (1.1)	2.86 (1.2)	2.00 (0.0)	2.60 (1.3)						
Completers	2.42 (1.1)	2.34 (1.0)	2.21 (1.0)	2.65 (1.2)						
**Alcohol consumption**^†^					.079	.92	.31	.58	.098	.91
Drop-outs	6.05 (5.6)	5.63 (6.3)	6.57 (6.7)	6.00 (3.4)						
Completers	5.34 (5.5)	5.00 (4.4)	4.97 (5.9)	5.92 (6.0)						

*ATYP – atypical symptom score ** BMI – Body Mass Index ^†^Alcohol consumption – drinks per week

### Remitted subjects

#### HDRS (see Table 3)

**Table 3:** Comparison of baseline variables (S.D) of subjects in remission (HDRS) with those who did not remit during the study

							***Analysis of variance***			
**Variable**					*Treatment group*		*Remitted group*		*Interaction*	

	**All**	**Exercise&Light**	**Exercise**	**Light**	**F**	**p**	**F**	**p**	**F**	**p**

**Age**					.11	.90	1.56	.22	.11	.89
Remitted	40.5 (10.5)	39.4 (11.6)	39.6 (11.2)	41.7(10.2)						
Not remitted	43.4 (9.0)	43.8 (8.0)	42.9 (9.6)	43.4 (9.7)						
**GSS**					1.59	.21	1.86	.18	.24	.79
Remitted	11.2 (4.7)	10.2 (5.2)	9.60 (5.0)	12.8 (3.9)						
Not remitted	12.6 (5.0)	12.8 (5.5)	11.4 (4.3)	13.5 (5.1)						
**HDRS**					3.09	.05	.76	.38	.11	.90
Remitted	14.2 (4.9)	16.3 (5.7)	11.0 (3.5)	14.0 (4.3)						
Not remitted	15.1 (6.4)	16.7 (8.1)	12.6 (3.7)	15.9 (6.2)						
**ATYP**					1.51	.23	.26	.61	.17	.84
Remitted	7.56 (5.0)	8.22 (4.7)	5.00 (3.5)	8.18 (5.6)						
Not remitted	7.76 (4.5)	8.27 (5.7)	6.69 (4.1)	8.28 (3.9)						
**BMI**					.63	.54	.088	.77	.51	.60
Remitted	24.5 (3.9)	24.8 (2.6)	22.4 (1.8)	25.3 (5.2)						
Not remitted	24.5 (4.4)	24.3 (5.4)	24.4 (3.8)	24.7 (4.4)						
**Sessions attended (%)**					.58	.56	8.66	**.004**	1.35	.27
Remitted	80.3 (15.8)	82.2 (10.0)	81.3 (27.2)	78.2(14.6)						
Not remitted	61.6 (30.1)	52.0 (33.6)	59.2 (31.1)	71.9(24.2)						
**Fitness**					.34	.57	.003	.96	.30	.59
Remitted	100.1 (13.7)	98.3 (16.5)	103.4 (6.8)	---						
Not remitted	100.6 (12.8)	100.5 (16.0)	100.7 (10.2)	---						
**BNSQ**										
*Initial insomnia*					3.83	**.03**	1.19	.28	1.87	.16
Remitted	2.32 (1.0)	2.22 (.97)	1.40 (.55)	2.82 (.98)						
Not remitted	2.42 (1.1)	2.71 (.99)	2.13 (1.2)	2.44 (.98)						
*Middle insomnia*					.43	.65	.00	.99	.47	.63
Remitted	3.36 (1.3)	3.22 (1.3)	3.20 (1.5)	3.55 (1.2)						
Not remitted	3.31 (1.2)	3.64 (.93)	3.00 (1.2)	3.33 (1.4)						
*Late insomnia*					1.37	.26	.69	.41	.90	.41
Remitted	2.04 (.94)	2.00 (1.0)	2.00 (1.0)	2.09 (.94)						
Not remitted	1.85 (.90)	1.93 (.83)	1.38 (.50)	2.22 (1.1)						
*Quality of sleep*					2.72	.07	.00	.99	.52	.60
Remitted	2.84 (1.2)	2.78 (1.1)	2.20 (1.1)	3.18 (1.3)						
Not remitted	2.71 (.99)	3.00 (.96)	2.31 (.70)	2.83 (1.2)						
**Alcohol consumption**					.17	.85	1.08	.30	1.29	.28
Remitted	5.08 (3.9)	6.78 (5.4)	3.80 (2.2)	4.27 (2.5)						
Not remitted	6.63 (6.8)	5.20 (5.4)	6.38 (7.5)	8.06 (7.2)						

Abbreviations as in Table 2

A total of 74 subjects (10 male, 64 female) were included in the analyses. Twenty-five subjects (3 male, 22 female, X^2 ^= .8) were considered remitted on the HDRS after the study period. Nine had been in the light & exercise group, 5 in the exercise group, and 11 in the light group (X^2 ^= .5). Thus, 20 of the subjects had received bright light therapy (X^2 ^= .3) and 14 had been in the exercise groups (X^2 ^= .5). Proportion of sessions attended had a significant impact on remission status (F = 8.66, p = .004). The BNSQ initial insomnia-variable was influenced by treatment group assignment (F = 3.83, p = .03).

#### SIGH-SAD (see Table 4)

**Table 4:** Comparison of baseline variables (S.D) of subjects in remission (SIGH-SAD total score) with those who did not remit during the study

							***Analysis of variance***			
**Variable**					*Treatment group*		*Remitted group*		*Interaction*	

	**All**	**Exercise&Light**	**Exercise**	**Light**	**F**	**p**	**F**	**p**	**F**	**p**

**Age**					.26	.77	.068	.80	.54	.58
Remitted	42.0 (10.5)	43.6 (11.4)	37.5 (6.4)	41.8 (11.2)						
Not remitted	42.0 (9.5)	40.1 (8.6)	41.7 (10.2)	43.6 (9.7)						
**GSS**					1.60	.21	5.23	**.03**	.17	.85
Remitted	10.62 (4.9)	10.3 (4.8)	7.00 (1.4)	11.8 (5.4)						
Not remitted	13.2 (4.7)	13.3 (5.7)	11.9 (4.3)	14.4 (4.1)						
**HDRS**					1.71	.19	1.07	.31	.093	.91
Remitted	14.5 (5.3)	15.3 (6.7)	11.5 (2.1)	14.5 (4.6)						
Not remitted	15.6 (6.2)	18.3 (7.9)	12.7 (3.8)	16.2 (5.7)						
**ATYP**					1.89	.16	2.68	.11	.52	.60
Remitted	7.71 (4.7)	8.29 (4.2)	3.50 (.71)	8.25 (5.4)						
Not remitted	8.88 (4.2)	10.3 (4.8)	7.76 (3.8)	8.84 (4.1)						
**BMI**					.63	.54	.57	.45	1.79	.18
Remitted	24.3 (2.9)	25.7 (2.5)	21.0 (1.4)	23.9 (2.8)						
Not remitted	24.7 (4.5)	23.4 (4.2)	24.6 (3.8)	25.6 (5.3)						
**Sessions attended (%)**					.64	.53	2.66	.11	.60	.55
Remitted	82.4 (14.9)	84.8 (6.34)	66.7 (47.1)	84.2 (8.68)						
Not remitted	65.3 (28.3)	59.0 (31.6)	64.3 (30.5)	70.9 (24.0)						
**Fitness**					1.06	.31	.12	.73	2.01	.17
Remitted	99.7 (16.5)	96.6 (17.7)	110.5 (2.1)	---						
Not remitted	101.4 (11.6)	102.7 (13.8)	100.5 (10.1)	---						
**BNSQ**										
*Initial insomnia*					2.46	.09	.69	.41	1.26	.29
Remitted	2.35 (.93)	2.00 (1.0)	1.50 (.71)	2.88 (.64)						
Not remitted	2.39 (1.0)	2.54 (.78)	2.12 (1.2)	2.53 (1.1)						
*Middle insomnia*					.19	.83	.88	.35	.707	.50
Remitted	3.18 (1.4)	3.00 (1.4)	3.00 (.00)	3.37 (1.6)						
Not remitted	3.47 (1.2)	3.92 (.95)	3.24 (1.2)	3.37 (1.3)						
*Late insomnia*					1.48	.24	.003	.96	.068	.94
Remitted	2.06 (.90)	2.00 (1.0)	1.50 (.71)	2.25 (.89)						
Not remitted	1.92 (.95)	1.85 (.99)	1.65 (.70)	2.21 (1.1)						
*Quality of sleep*					1.87	.16	.21	.65	1.35	.27
Remitted	2.82 (1.1)	2.43 (.98)	2.00 (.00)	3.38 (1.1)						
Not remitted	2.76 (1.1)	2.92 (1.2)	2.53 (.87)	2.84 (1.3)						
**Alcohol consumption**					.059	.94	1.49	.23	.26	.77
Remitted	4.06 (2.8)	4.43 (3.3)	4.50 (3.5)	3.63 (2.4)						
Not remitted	6.50 (6.1)	5.36 (5.7)	6.76 (7.0)	7.11 (5.8)						

Abbreviations as in Table 2

Sixty-seven subjects (8 male, 59 female) were included in the analyses. After the trial, sixteen of them (1 male, 15 female, X^2 ^= .4) were considered to be in remission on the SIGH-SAD-scale. Seven subjects had been in the light & exercise group, 1 in the exercise group, and 8 in the light group (X^2 ^= .08). Fifteen subjects (94%) had been in groups with bright light exposure (X^2 ^= .03), and eight (50%) in the exercise groups (X^2 ^= .4). In the ANOVA-models, remission was significantly influenced only by the GSS (F = 5.23, p = .03).

## Discussion

### Treatment response

The results of this study confirm earlier findings from light and exercise trials that these interventions are well tolerated and effective. Considering the study population was not a clinical one, but consisted of volunteers not suffering from any major mental or physical disorder, the NNT for light therapy was high: 4 subjects need to be treated for one subject to respond, as assessed with both the HDRS and the SIGH-SAD-SR. The overall response rate, on all the scales, was about 50 %.

Initial insomnia at the start of the study was an independent variable predicting good response, both on the ATYP and the SIGH-SAD-SR. Disorders of sleep are common in depressive states, and also in the general population [27]. The mean GSS of our study subjects was relatively high, suggesting that they had a marked degree of seasonal variation in mood and behaviour and might be predisposed to desynchrony between the sleep-wake cycle and circadian rhythms in winter. It is probable that entrainment of internal clocks by environmental stimuli is impaired in depression. Light and exercise are both capable of entraining the circadian rhythms, which could be one, but not the only mechanism of action reflected externally as improved sleep and mood [28]. However, a limitation of our study was that we did not measure circadian rhythms.

Ageing changes sleep-wake habits. This may be due to a deteriorating impact of light with age on the flexibility of the internal clock [29]. No studies comparing the young and the old on the benefits of bright light therapy have been published to our knowledge. Age has not been a significant factor in trials of bright light therapy. Baehr et al. have examined the circadian phase-shifting effects of exercise in two age groups (20–32 and 55–73 years), and no significant differences were found [30]. However, our hypothesis that the older age group would benefit from this kind of intervention was not supported; in fact, the opposite occurred. One explanation for this is the frequency of the intervention: two times a week may be a signal powerful enough to entrain circadian rhythms for younger subjects, but not for older subjects, even when light and exercise interventions are combined.

We found that even moderate consumption of alcohol (>7 drinks per week) predicted a poorer response on all the assessed scales. One explanation to this might be the negative effect of alcohol on circadian rhythms and especially on sleep [31,32].

### Dropout from the study

The dropout rate was relatively low (combined 21%), and did not differ significantly between the treatment conditions. There seemed to be a trend towards subjects in groups receiving bright light therapy adhering to the study more closely than subjects in the exercise group. Subjects might have felt bright light therapy is a novel, more attractive treatment option than plain, 'old-fashioned' exercise. We tried to minimise this problem by emphasising 'exercise trial' in leaflets provided to possible volunteers. It was not possible to avoid this problem altogether, as reflected by those four subjects who dropped out of the study after hearing they had been randomised into the exercise group. When these four subjects, now counted as dropouts, are excluded, the dropout rates of the light & exercise and exercise groups are virtually identical, and similar to the rates reported previously [20]. The dropout rate in the light group was considerably lower than in the exercise groups, but the difference failed to reach statistical significance. Subjects possibly experienced bright light exposure without the strenuous exercise very comfortable, which is understandable.

An interesting finding is that completers were in a better physical condition in the pre-intervention fitness test than were dropouts. Regular exercising may be a representation of self-motivation trait, which would increase adherence to a therapeutic exercise program [33]. A major limitation is of course that the subjects in the bright light group did not perform the fitness test. This had to be omitted from the study protocol for economical reasons.

### Remitted subjects

Using the strict remission criteria yielded few results. Attendance to treatment sessions did predict remission on the Hamilton scale (but not the SIGH-SAD total score), but post-hoc analyses showed that this was caused by dropouts, which were automatically labeled as treatment failures.

A lower GSS was predictive of remission on the SIGH-SAD total score. Again, post-hoc analyses showed this effect was caused by dropouts with higher than average scores.

In previous research on the use of light therapy, atypical depressive symptoms have been predictive of treatment response and remission. This effect was not seen in the present study. We did separate analyses for hypersomnia, hyperphagia, increased appetite / carbohydrate craving, and reduced vitality, but none of these individual atypical symptoms predicted remission either.

### Assessment

The study subjects were not patients with clinically diagnosed depression, but volunteers with varying degrees of depressive symptoms. This poses a major question of definition of treatment response and remission, and also measurement of depressive symptoms, e.g. using scales originally designed for the follow-up of depressed in-patients [34]. Our primary focus in planning this study was practical: to find an intervention that would benefit the public at large. Study subjects were not a random sample of population, but volunteers invited through occupational health centres, and free of pre-existing, diagnosed or medicated mental illness. However, we decided to use established methods for the assessment of depressive symptoms. In their systematic review on exercise studies, Lawlor and Hopker demand the use of dichotomous outcomes, arguing them to be more understandable and more important outcomes in clinical terms [7]. We agree, and use the concepts of response and remission in this study. The cut-offs selected were based on previous studies [35], done on depressed patients. The application of these criteria to a trial with healthy subjects may seem artificial, but we feel this increases the clinical meaningfulness of the results.

### Future research

More studies, with expanded methodology, are clearly needed to shed light on the individual factors separating responders and non-responders in exercise trials, with or without bright light. Interesting research subjects would be circadian phenotyping (with Morning-Eveningness Questionnaire; [36]), and motor activity measurement. Pre-treatment expectations should be assessed, to estimate the possible placebo-effect.

## Conclusions

We investigated the effect of bright light and exercise on depressive symptoms in working-age men and women, free from mental disorder and/or psychotropic medication. We found that problems with sleep, especially initial insomnia, may predict a good response to treatment using combined light and exercise. Regular intake of alcoholic beverages (over 7 drinks per week) seems to have an opposite effect. Bright light exposure and physical exercise, even in combination, seem to be well tolerated and effective on depressive symptoms, but more research is needed to confirm these findings.

## Competing interests

None declared.

## Authors' contributions

SL, TP, and JL planned the study protocol and supervised the study. JH planned the statistical analyses. SL, TP, and JH analysed the data. SL and TP wrote the manuscript, which was commented by JH and JL. All authors have read and approved the final manuscript.

## Pre-publication history

The pre-publication history for this paper can be accessed here:


